# Macrophage Subset Sensitivity to Endotoxin Tolerisation by *Porphyromonas gingivalis*

**DOI:** 10.1371/journal.pone.0067955

**Published:** 2013-07-15

**Authors:** Andrew D. Foey, StJohn Crean

**Affiliations:** 1 School of Biomedical and Biological Sciences, University of Plymouth, Plymouth, United Kingdom; 2 Faculty of Health and Social Work, University of Central Lancashire, Preston, United Kingdom; University of Florida, College of Dentistry & The Emerging Pathogens Institute, United States of America

## Abstract

Macrophages (MΦs) determine oral mucosal responses; mediating tolerance to commensal microbes and food whilst maintaining the capacity to activate immune defences to pathogens. MΦ responses are determined by both differentiation and activation stimuli, giving rise to two distinct subsets; pro-inflammatory M1- and anti-inflammatory/regulatory M2- MΦs. M2-like subsets predominate tolerance induction whereas M1 MΦs predominate in inflammatory pathologies, mediating destructive inflammatory mechanisms, such as those in chronic *P.gingivalis* (PG) periodontal infection. MΦ responses can be suppressed to benefit either the host or the pathogen. Chronic stimulation by bacterial pathogen associated molecular patterns (PAMPs), such as LPS, is well established to induce tolerance. The aim of this study was to investigate the susceptibility of MΦ subsets to suppression by *P. gingivalis*. CD14^hi^ and CD14^lo^ M1- and M2-like MΦs were generated *in vitro* from the THP-1 monocyte cell line by differentiation with PMA and vitamin D_3_, respectively. MΦ subsets were pre-treated with heat-killed PG (HKPG) and PG-LPS prior to stimulation by bacterial PAMPs. Modulation of inflammation was measured by TNFα, IL-1β, IL-6, IL-10 ELISA and NFκB activation by reporter gene assay. HKPG and PG-LPS differentially suppress PAMP-induced TNFα, IL-6 and IL-10 but fail to suppress IL-1β expression in M1 and M2 MΦs. In addition, *P.gingivalis* suppressed NFκB activation in CD14^lo^ and CD14^hi^ M2 regulatory MΦs and CD14^lo^ M1 MΦs whereas CD14^hi^ M1 pro-inflammatory MΦs were refractory to suppression. In conclusion, *P.gingivalis* selectively tolerises regulatory M2 MΦs with little effect on pro-inflammatory CD14^hi^ M1 MΦs; differential suppression facilitating immunopathology at the expense of immunity.

## Introduction

Chronic periodontitis (CP) is a persistent inflammatory condition of the periodontal tissues resulting in destruction of the periodontium which, if left untreated, could result in tooth loss. CP results as a consequence of the host inflammatory response to persistent microbial challenge represented by a dysbiotic biofilm in which *Porphyromonas gingivalis* (PG) is an important member [1]–[3]. PG is an intracellular oral mucosal pathogen which evades recognition and uptake by neutrophils, infecting oral epithelial cells, fibroblasts and underlying dendritic cells and macrophages (MΦs) [4]–[6]. Clearance of such intracellular pathogens would necessitate cell mediated immunity, involving Th_1_ subset cells. *Porphyromonas gingivalis* LPS (PG-LPS) however, predominantly induces Th_2_-mediated humoral responses to extracellular pathogens; hence immune-deviation towards a non-clearing response is integral to pathogen persistence [7]. PG-LPS also possesses low endotoxin activity and targets TLR2, at the expense of the traditional LPS receptor, TLR4, although *P. gingivalis* strains exhibit differential structural LPS formats to and, as a consequence, differential utilisation of both TLR2 and TLR4 [8]. Thus, PG subverts both adaptive and innate immune function to survive in oral mucosal tissue.

Immune subversion can be achieved by both immunomodulatory and immunosuppressive mechanisms. PG-LPS is able to induce endotoxin tolerance (ET) in MΦs; ET was first characterised by LPS pre-exposure rendering innate immune cells refractory to subsequent endotoxin challenge, reviewed in [9]. ET would appear to be both beneficial and harmful to host and pathogen alike; suppressing harmful over-exuberant tissue-destructive pro-inflammatory responses, manifestation of sepsis, autoimmunity and cancer in the host [10], whereas, simultaneously, suppresses protective inflammatory responses mounted against the oral pathogen. Oral mucosal MΦs are important to ET; their differentiation and activation status determining whether the mucosal environment is beneficial to the host tissue or pathogen. PG modulates host cell function in order to facilitate its own survival [11], [12]. Upon LPS recognition, this pathogen induces an inflammatory response modulated by a wide range of inflammatory molecules. Of interest however, is that PG only weakly induces inflammatory cytokines, favouring an insufficient clearing response, bacterial proliferation and persistence. The cytokine production in response to this expanded bacterial number contributes to localised tissue destruction characteristic of chronic periodontitis [13]–[15].

MΦs densely populate oral mucosa, responding to *P.gingivalis* by producing pro-inflammatory cytokines such as TNFα, IL-1α, IL-1β, IL-18, IL-18R, IL-18RAcp, IL1F9, IL-6, LIF, IL-12, IL-8, CCL2, CXCL10, MCP-1 and IL-32. Conversely, expression of anti-inflammatory cytokines (eg. IL-10) are induced, but at much lower levels compared to pro-inflammatory cytokines [16]. This profile is suggestive of a MΦ resembling the M1 pro-inflammatory subset. In the context of non-infected homeostatic oral mucosal tissue, the cytokine effector phenotype resembles the anti-inflammatory/regulatory M2 subset, reviewed in [17]. PG-LPS-induced MΦ cytokine profiles are indeed suggestive of M1 subset association with pro-inflammatory pathology whereas M2 MΦs are associated with regulatory/homeoatatic conditions. M1 MΦs are activated by LPS through TLR4, inducing NFκB -dependent pro-inflammatory cytokines. M2 MΦs however, exhibit similar PRR expression and a different cytokine profile where pro-inflammatory cytokine expression is relatively lower compared to M1 MΦs, reviewed in [18]. In addition, MΦ subsets exhibit a differential NFκB -dependency; where M1 activity is dependent on p65/p50 NFκB and M2 on p50/p50 NFκB [19]–[21], determining responses as activatory/pro-inflammatory or tolerogenic/anti-inflammatory. MΦ tolerance can be induced by several different mechanisms: these include down-regulation of PRRs, induction of suppressive cytokines (TGFβ and IL-10) and pro-inflammatory cytokine analogues, shedding of cytokine receptors and PRRs and induction of endogenous inhibitors to PRR-mediated signalling such as Tollip, Myd88s, SARM, sTLRs, sCD14 and SIGIRR.

MΦs express both TLR2 and TLR4; responses to their respective PAMPs, lipopeptides and LPS are optimised by association with the co-receptor molecule, CD14, driving potent inflammatory responses characterised by high levels of the pro-inflammatory cytokines, TNFα, IL-1β, IL-6, IL-8. Indeed, CD14 gene polymorphisms are associated with inflammatory periodontal disease, where CD14^hi^ expression is indicative of higher levels of inflammation [22]. CD14 expression is partially predictive of mucosal MΦ effector phenotype: CD14^lo^ MΦs produce anti-inflammatory/regulatory cytokines (TGFβ and IL-10) and low levels of pro-inflammatory cytokines [23]. As such, mucosal MΦs, existing in a non-pathogenic and homeostatic state, resemble the M2 MΦ phenotype. CD14^hi^ MΦs, on the other hand, produce high levels of pro-inflammatory- and low levels of regulatory- cytokines: resembling M1 MΦs, readily activated by PAMPs which, if uncontrolled, drive chronic inflammatory pathology. Thus, mucosal MΦ effector phenotype (inflammatory vs regulatory) may be controlled by regulation of TLR and CD14 expression. Of significance to control of effector phenotype is the observation that gingipains, released from outer membrane vesicles of *P.gingivalis*, have been described to cleave CD14 from the membrane surface [24]. Such a mechanism can suppress MΦ inflammatory responses (LPS hypo-responsiveness) and represents another tolerogenic response associated with ET.

The relevance of ET in the pathology of CP is the subject of intense research efforts. ET may benefit both the host and pathogen; tolerance would normally be viewed as beneficial in the context of a destructive inflammatory pathology, whereas in the case of PG, ET may favour pathogen persistence. PG-LPS is predominantly recognised by TLR2, instead of TLR4. In CP, both TLR2^+^ and TLR4^+^ monocytes are recruited into the gingival lamina propria whereas, concurrently, in diseased human CP gingiva, mucosal tissue was generally tolerised where TLR2, TLR4, TLR5 and MD-2 expression was down-regulated. Functional studies substantiated these results, PG-LPS pre-treatment of monocytes suppressed subsequent PG-LPS stimulation of both pro-inflammatory (TNFα, IL-1β, IL-6, IL-8) and anti-inflammatory cytokines (IL-10) [25]. The aim of this study was thus two-fold: to investigate whether *Porphyromonas gingivalis* differentially modulates cytokine production in the pro-inflammatory M1-like MΦ subset in comparison to the anti-inflammatory/regulatory M2-like subset and to expand on current understanding of *P. gingivalis*-induced endotoxin tolerance in the context of these functionally disparate MΦ subsets, relevant to mucosal MΦ effector function.

## Materials and Methods

### Monocyte and macrophage **(**MΦ**)** culture

The human monocytic cell line, THP-1, was obtained from ECACC and routinely used between passages 7 and 25. THP-1 cells were maintained in RPMI-1640 medium supplemented with 10% v/v foetal calf serum, 2 mM L-glutamine,100 U/ml penicillin and 100 µg/ml streptomycin (Lonza, Wokingham, UK), here on referred to as R10 medium. The THP-1 NFκB reporter cell lines THP-1Blue (CD14^lo^) and THP-1Blue-CD14 (CD14^hi^) (Autogen Bioclear, Calne, UK) were maintained in R10 medium in the presence of the selection antibiotics, zeocin (200 µg/ml) only (CD14^lo^) or 200 µg/ml zeocin and 10 µg/ml blastocidin (CD14^hi^) (Autogen Bioclear, Calne, UK). Stable expression status of membrane-associated CD14 as either CD14^lo^ or CD14^hi^ was routinely checked by flow cytometry. Cells were plated out at a density of 1×10^5^cells/well in R10 medium in 96 flat-bottomed well tissue culture plates (monocyte cultures). Pro-inflammatory (M1-like) MΦs and anti-inflammatory (M2-like) MΦs were generated by monocyte differentiation in the presence of 25 ng/ml PMA or 10 nM 1,25-(OH)_2_-Vitamin D_3_ (Sigma-Aldrich, Poole, UK) for 3 and 7 days, respectively [26].

### Bacteria and pathogen associated molecular patterns **(**PAMPs**)**

Bacterial products were obtained from Autogen Bioclear, Calne, UK. *P. gingivalis* strain ATCC 33277 was originally isolated from human gingival sulcus and obtained from the American Type Culture Collection. Due to the ability of *P.gingivalis* to induce inflammatory factors via membrane receptors and to invade mucosal cells by phagocytosis, the effects of *Porphyromonas gingivalis* lipopolysaccharide (PG-LPS) were compared to those obtained for whole bacterial cells, heat-killed *Porphyromonas gingivalis*, HKPG (in the absence of any secreted bacterial products). PG-LPS was extracted by successive enzymatic hydrolysis and purification by Phenol-TEA-DOC protocol, described in [27]. HKPG were prepared by heating a bacterial suspension of *P.gingivalis* to 120°C for 30 minutes followed by several washes in endotoxin-free water. Peptidoglycan (PGN) was purchased from Sigma-Aldrich, Poole, Dorset, UK.

### Activation of monocyte and macrophage cytokine production

THP-1, THP-1(CD14^lo^) and THP-1(CD14^hi^)-derived M1- and M2-like MΦs were stimulated by the bacterial pathogen associated molecular patterns (PAMPs); 100 ng/ml PG-LPS, 1×10^7^cells/ml HKPG and 10 µg/ml of the TLR2-ligand, lipoteichoic acid (LTA) (Autogen Bioclear, Calne, UK) and cultured for 18 hours (determined as optimal PAMP concentration and time period for expression of all the pro-inflammatory cytokines TNFα, IL-1β and IL-6, data not shown). Supernatants were then harvested and either used immediately for colorimetric analysis of NFκB activity or alternatively, stored at −20°C until required for cytokine assay by sandwich ELISA.

### Tolerisation by pre-incubation with *Porphyromonas gingivalis* PAMPs, LTA and PGN

THP-1, THP-1(CD14^lo^) and THP-1(CD14^hi^)-derived M1- and M2-like MΦs were pre-treated for 24 hours with either 100 ng/ml PG-LPS, 1×10^7^cells/ml HKPG, 10 µg/ml LTA or 10 µg/ml PGN (determined as the optimal concentration and time duration for tolerisation, data not shown) or R10 medium alone (tolerisation negative control). Pre-stimulus culture medium was carefully removed, after which MΦs were washed in fresh R10 prior to stimulation for a further 18 hours at 37°C/5% CO_2_. MΦs were either stimulated with 100 ng/ml PG-LPS, 1×10^7^cells/ml HKPG, 10µg/ml LTA or R10 medium alone (stimulation negative control). After this stimulation period, supernatants were harvested and either used immediately for colorimetric analysis of NFκB activity or stored at −20°C until required for cytokine assay by sandwich ELISA. To demonstrate a physiologically-relevant tolerisation; after stimulation or tolerisation protocols, MΦ viability was routinely checked by either MTT assay or trypan blue exclusion. No significant reductions in viability were observed for PAMPs used in this study, viability was routinely >85%.

### Cytokine measurement

Cytokines; TNFα, IL-1β, IL-6 and IL-10 were analysed by sandwich ELISA using capture and detection antibodies commercially available from R&D Systems UK Ltd., Abingdon and BD-Pharmingen, Oxford, UK. Protocols were followed according to manufacturer's instructions and compared to standard curves, between the range of 7 to 5,000 pg/ml, using the international standards available from NIBSC, Potter's Bar, UK. Colorimetric development was measured spectrophotometrically by an OPTIMax tuneable microplate reader at 450 nm and analysed by Softmax Pro version 2.4.1 software (Molecular Devices Corp., Sunnyvale, CA, USA).

### NFκB activity measurement

NFκB activity was measured using a colorimetric reporter gene assay for secreted embryonic alkaline phosphatase (SEAP) associated with the stably-transfected reporter gene cell lines, THP-1Blue (CD14^lo^) and THP-1Blue-CD14 (CD14^hi^). Briefly, at conclusion of the experiment, conditioned medium was harvested and incubated with Quantiblue colorimetric reagent (Autogen Bioclear, Calne, UK) for 30 minutes at 37°C/5% CO_2_. Colorimetric development was then measured spectrophotometrically by an OPTIMax tuneable microplate reader at 620 nm and analysed by Softmax Pro version 2.4.1 software. The resulting colour development was directly proportional to the reporter gene SEAP expression and hence NFκB activity.

### Statistical analysis

Measure of statistical significance was analysed using a balanced analysis of variance (General Linear Model, Minitab version 16) followed by a multiple comparison test (LSD, least significant difference). Significance was set at p<0.05 (*p<0.05, **p<0.01 and ***p<0.001).

## Results

### PG-LPS and HKPG induce separate pro-inflammatory cytokine profiles in M1 and M2 MΦs

**Figure 1:**
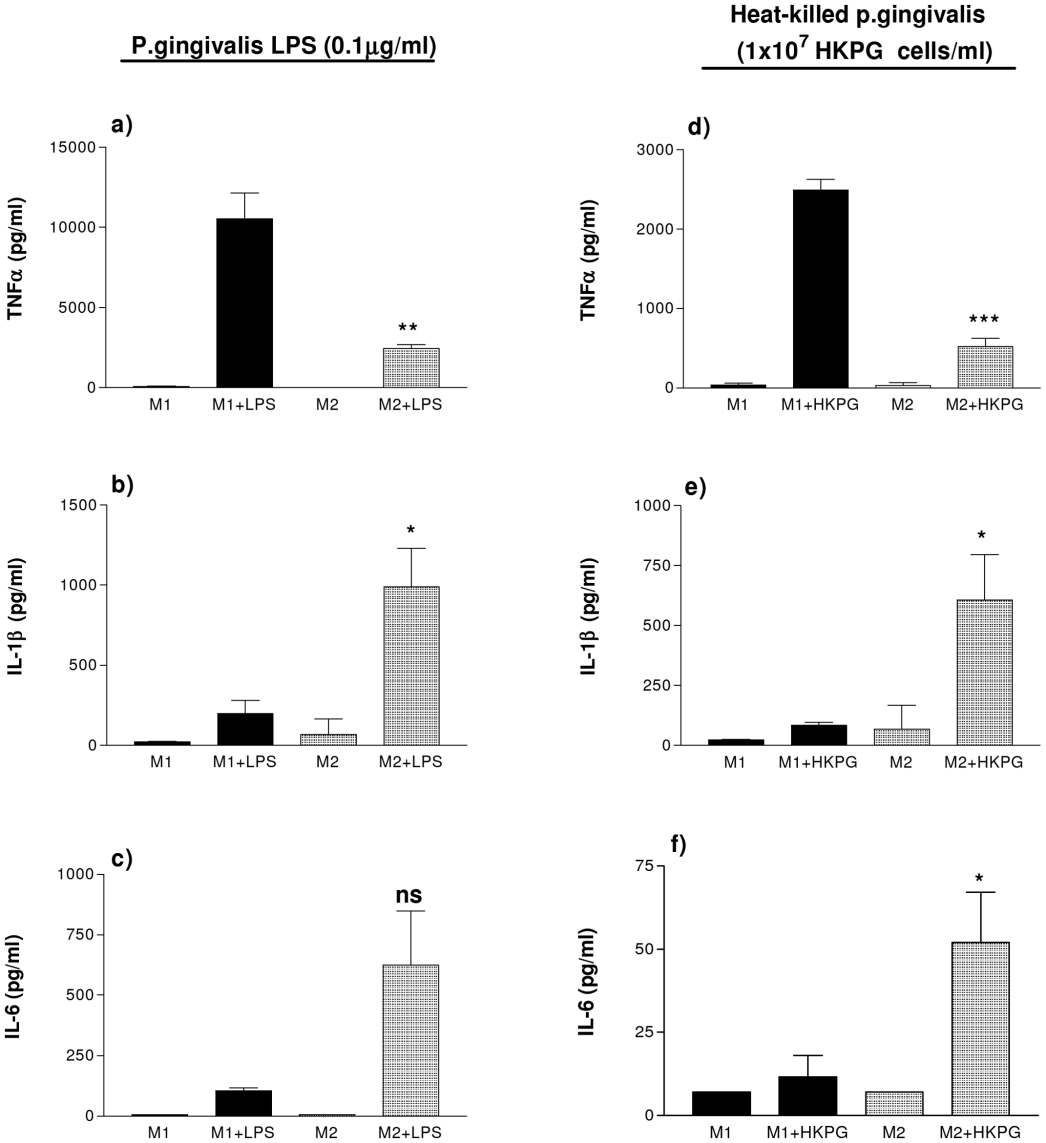
M1 & M2 MΦs display differential cytokine profiles in response to PG-LPS and HKPG. THP-1-derived M1 and M2 MΦs were generated by differentiating THP-1 monocytes with either 25 ng/ml phorbol 12-myristate 13-acetate (PMA) for 3 days or 10 nM 1,25-(OH)_2_ vitamin D_3_ for 7 days, respectively. M1 (bold) and M2 (shaded) MΦ subsets were stimulated with either 100 ng/ml PG-LPS (a, b and c) or 1×10^7^ cells/ml HKPG (d, e and f). Cytokine production is expressed as the mean ± SD in pg/ml for TNFα (a & d), IL-1β (b & e) and IL-6 (c & f). Data displayed represents triplicate samples for n = 3 replicate experiments. Significant differences in cytokine production between activated M1 and M2 MΦs are indicated as *p<0.05, **p<0.01, ***P<0.001 and ns, not significant.

Upon stimulation M1 and M2 MΦ subsets produce different cytokine profiles; M1 MΦs exhibit a predominantly pro-inflammatory cytokine profile whereas M2 MΦs express a more anti-inflammatory or regulatory profile. This experiment was undertaken to establish whether M1 and M2 MΦs responded similarly to challenge with the oral pathogen, *P.gingivalis*. Indeed, PG induced distinct cytokine profiles in M1 and M2 MΦs. Stimulation of these MΦ subsets was comparable, however, when stimulated by either HKPG or PG-LPS: PG-LPS induced M1 expression of the pro-inflammatory cytokines TNFα, IL-1β and IL-6 at a ratio of 99∶2∶1. On the other hand, PG-LPS induced a TNFα: IL-1β: IL-6 ratio in M2 MΦs of 4∶2∶1, where the cytokine expression between these two MΦ subsets was significant to p = 0.0098 for TNFα, p = 0.046 for IL-1β and p = 0.062 for IL-6 (Figure 1a, b & c). A similar cytokine profile was observed when M1 and M2 MΦs were stimulated by HKPG. HKPG induced an M1 expression profile of 249∶8∶1 and 10∶12∶1 in M2 MΦs, where the cytokine expression between these two MΦ subsets was significant to p = 0.0008 for TNFα, p = 0.044 for IL-1β and p = 0.033 for IL-6 (Figure 1d, e & f). As a consequence of heterogeneity of CD14 expression on M1- and M2-like macrophages, IL-10 secretion was not routinely detectable above the lower limit of detection of the IL-10 ELISA, and as such was not presented in this figure. IL-10 secretion however, was detectable when examining a more homogenous CD14^hi^ and CD14^lo^ macrophage population (refer to later stable transfectant data figures and tables).

In addition, these THP-1 derived macrophage subsets both display a differential response towards the enteropathic *E. coli* K12 LPS and the oral pathogenic *P. gingivalis* LPS. In agreement with other studies [8], PG-LPS exhibits low endotoxin activity when compared with the same concentration of K12-LPS. In the case of M1 and M2 MΦs, endotoxin activity was determined by the strength of induction of TNFα secretion; PG-LPS resulted in 13% TNFα induction in M1s compared to K12-LPS whereas in the case of M2 MΦs, PG-LPS resulted in 25% TNFα induction. This may be consistent with PG-LPS utilisation of TLR2, as TNFα induction was closer in amplitude to that of the TLR2 agonist, LTA (see table 1).

**Table 1:** PG-LPS exhibits weak endotoxin activity in THP-1-derived macrophages.

Treatment	M1-like macrophages	M2-like macrophages
**Control**	9.0±7.6	7.0±0.0
**LTA**	347±92	1329±141
**K12-LPS**	5107±775	2857±480
**PG-LPS**	643±79	704±36

THP-1-derived M1-like (PMA) and M2-like (Vit D_3_) MΦs were either unstimulated (control) or stimulated with 100 ng/ml PG-LPS, 100 ng/ml *E. coli* K12 LPS (TLR4) or 10 µg/ml LTA (TLR2) for 18 hours. Endotoxin activity was investigated by TNFα secretion, presented as the mean ± SD in pg/ml. Data displayed is representative of triplicate samples for n = 3 replicate experiments.

### *P. gingivalis* differentially suppresses M1 and M2 MΦ pro-inflammatory cytokines

**Figure 2:**
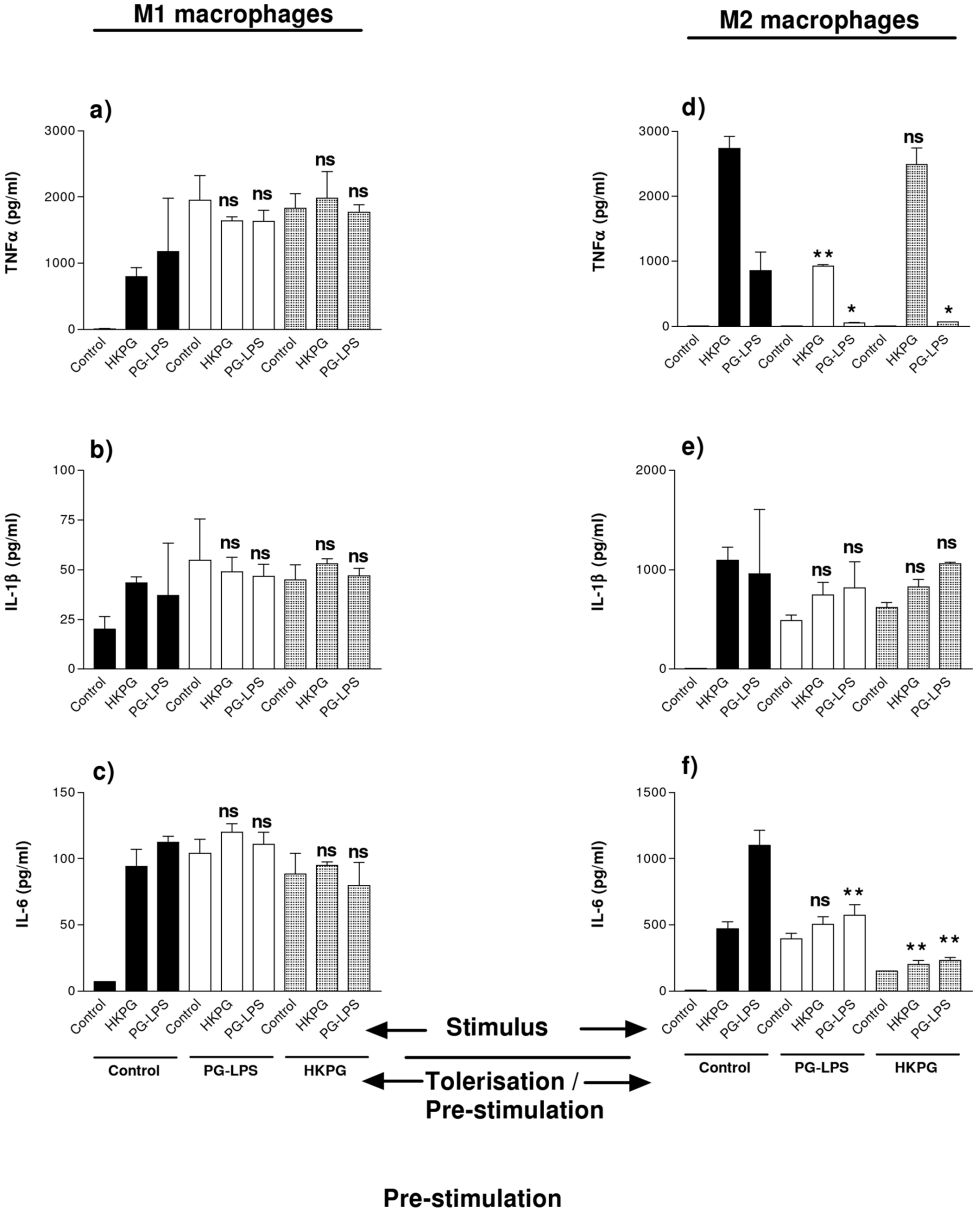
*P.gingivalis* differentially suppresses M1 & M2 MΦ cytokines. M1 (a, b & c) and M2 (d, e & f) MΦ subsets were pre-stimulated (tolerised) with either 100 ng/ml PG-LPS (unshaded) or 1×10^7^ cells/ml HKPG (shaded) for 24 hours prior to stimulation with PG-LPS or HKPG and incubated for a further 18 hours (untolerised controls indicated in bold). Cytokine production is expressed as the mean ± SD in pg/ml for TNFα (a & d), IL-1β (b & e) and IL-6 (c & f). Data displayed represents triplicate samples for n = 3 replicate experiments. Significant effects compared to the un-tolerised stimulus control (bold) for the indicated MΦ subset are indicated as *p<0.05, **p<0.01 and ns, not significant.

Macrophage challenge with *Porphyromonas gingivalis* (PG-LPS and HKPG) differentially suppresses MΦ subset cytokine production upon stimulation with the same pre-treatment challenges. Pre-treatment of M1 pro-inflammatory MΦs fails to suppress TNFα, IL-1β and IL-6 when later challenged by PG-LPS and HKPG (see figure 2a, b & c). M2-like MΦs, on the other hand, were sensitive to tolerance induction. PG-LPS pre-treatment strongly suppressed M2 production of TNFα, upon stimulation with either PG-LPS (reduced by 94%, p = 0.0383) or HKPG (reduced by 66%, p = 0.0032) (See fig. 2d). Pre-treatment with HKPG partially suppressed TNFα production stimulated by HKPG (reduced by 9%, p = 0.258) but clearly suppressed PG-LPS induced TNFα (reduced by 92%, p = 0.0433) (see fig. 2d). In addition to PG-LPS tolerising TNFα production to PG-LPS stimulation and HKPG tolerising HKPG stimulation, these data also demonstrate a level of cross-tolerisation between HKPG and PG-LPS with respect to TNFα production by M2 MΦs. M2 production of IL-1β, however, failed to show any significant suppression in response to both pre-treatment and stimulation by either HKPG or PG-LPS (fig. 2e). IL-6 production, on the other hand, was partially suppressed, dependent on pre-stimulation and challenge stimulus. Pre-treatment with HKPG partially suppressed IL-6 production stimulated by HKPG (reduced by 57%, p = 0.0067) but clearly suppressed PG-LPS induced IL-6 (reduced by 79%, p = 0.0078) (see fig. 2f). Pre-treatment with PG-LPS failed to suppress IL-6 production stimulated by HKPG, but clearly suppressed PG-LPS induced IL-6 (by 48%, p = 0.0013) (fig. 2f).

### PG-LPS and HKPG induction of pro-inflammatory cytokine profiles in M1 and M2 MΦs is CD14-dependent

**Figure 3:**
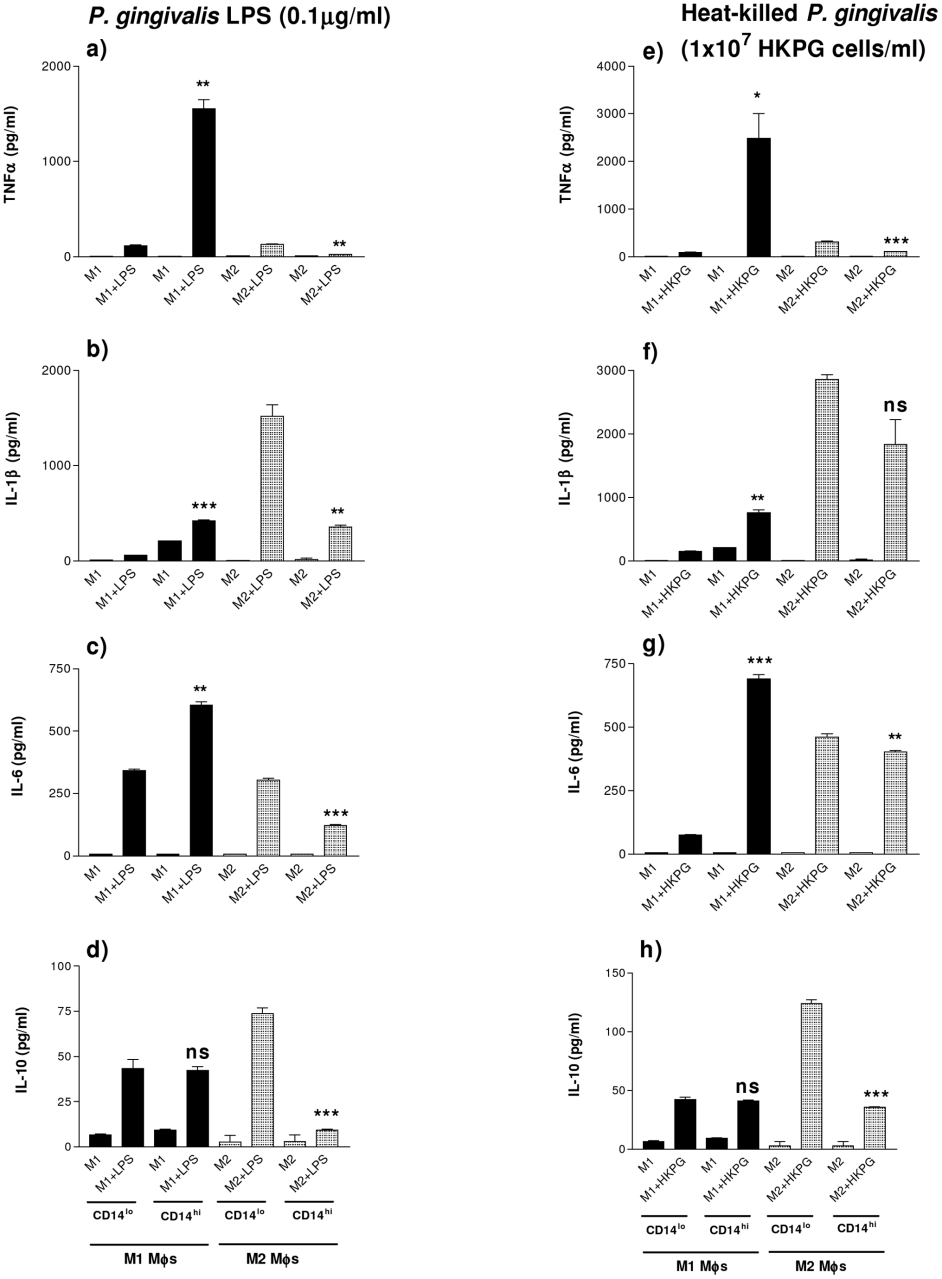
PG-LPS and HKPG induction of M1 and M2 MΦ pro-inflammatory cytokines are CD14-dependent. THP-1-derived CD14-high- and CD14-low-expressing (CD14^hi^ and CD14^lo^) M1 and M2 MΦs were generated by differentiating CD14^+^ and CD14^−^ stable transfectant THP-1-blue monocytes with either 25 ng/ml phorbol 12-myristate 13-acetate (PMA) for 3 days or 10 nM 1,25-(OH)_2_ vitamin D_3_ for 7 days, respectively. CD14^hi^/CD14^lo^ M1 (bold) and M2 (shaded) MΦ subsets were stimulated with either 100ng/ml PG-LPS (a, b, c & d) or 1×10^7^ cells/ml HKPG (e, f, g & h). Cytokine production is expressed as the mean ± SD in pg/ml for TNFα (a & e), IL-1β (b & f), IL-6 (c & g) and IL-10 (d & h). Data displayed represents triplicate samples for n = 3 replicate experiments. Significant differences in cytokine production between activated CD14^hi^ and CD14^lo^ MΦs are indicated as *p<0.05, **p<0.01, ***P<0.001 and ns, not significant.

In the homeostatic, regulatory mucosal environment, mucosal MΦs exhibit an M2-like phenotype characterised by a regulatory cytokine profile and the absence of surface markers such as CD14 and CD89. The inflammatory environment results in recruitment of CD14^+^ monocytes, which differentiate to a CD14^hi^ inflammatory phenotype, resembling M1-like MΦs. In an attempt to mimic mucosal MΦs in both homeostatic and inflamed tissue, the pro-inflammatory cytokine profiles of M1 and M2 MΦs, in response to stimulation by HKPG and PG-LPS, was investigated for CD14^lo^ and CD14^hi^ transfectant MΦs. These CD14^lo^ and CD14^hi^ MΦs exhibited subtle changes in pro-inflammatory cytokine expression: M1 & M2 CD14^lo^ MΦs display different amplitudes of cytokine expression compared with CD14^hi^ MΦs in response to PG-LPS and HKPG. When stimulated by PG-LPS, M1 CD14^hi^ MΦs expressed higher levels of TNFα, IL-1β and IL-6 at a ratio of 2.6∶0.7∶1.0, respectively) compared to M1 CD14^lo^ MΦs which produced a ratio of 0.34∶0.17∶1.0, where the cytokine expression between these two macrophage subsets was significant to p = 0.0012 for TNFα, p = 0.0001 for IL-1β and p = 0.0019 for IL-6 (Figure 3a, b & c). Conversely, the opposite expression was observed with M2 MΦs: M2 CD14^lo^ MΦs expressed greater levels of TNFα, IL-1β and IL-6 at a ratio of 0.43∶5.0∶1.0 respectively compared to that of M2 CD14^hi^ MΦs 0.21∶3.0∶1.0, where the cytokine secretion between these two MΦ subsets was significant to p = 0.0013, p = 0.0039, and p = 0.0002, respectively) (figure 3a, b and c). This same cytokine profile was reproduced for CD14^hi^/CD14^lo^ M1 and M2 subsets when stimulated with HKPG, where CD14^hi^ M1 produced higher levels of TNFα (p = 0.0147), IL-1β (p = 0.004) and IL-6 (p = 0.0003) when compared to CD14^lo^ M1 MΦs and conversely, CD14^lo^ M2 MΦs produced higher levels of TNFα (p = 0.0007), IL-1β (p = 0.061) and IL-6 (p = 0.0033) compared to CD14^hi^ M2 cells (figure 3e, f and g). Finally, a similar profile of production for the anti-inflammatory cytokine, IL-10, was observed. CD14^lo/hi^ M1 MΦs produced similar low levels when stimulated by PG-LPS (43±5 and 42±2 pg/ml) and HKPG (42±2 and 41±1 pg/ml), respectively. M2 regulatory MΦs however, exhibited a differential induction of IL-10 in response to PG stimulation. CD14^lo^ M2 MΦs produced 74±3 and 124±4 pg/ml in response to PG-LPS and HKPG, whereas CD14^hi^ M2s produced appreciably lower levels of IL-10 (9±1 and 36±1 pg/ml), respectively (figure 3d and h).

As with the case of non-transfected THP1-derived macrophage subsets, PG-LPS demonstrated a weak endotoxin activity when compared to *E. coli* K12-LPS-induction of TNFα in stably transfected CD14^hi/lo^ M1/M2 macrophages. PG-LPS endotoxin activity was 5%, 16%, 8% and 4% that of K12-LPS induction of TNFα by CD14^lo^ M1, CD14^hi^ M1, CD14^lo^ M2 and CD14^hi^ M2, respectively (see table 2).

**Table 2:** PG-LPS exhibits weak endotoxin activity in CD14^hi/lo^ M1/M2 macrophages.

Treatment	CD14^lo^ M1	CD14^hi^ M1	CD14^lo^ M2	CD14^hi^ M2
**Control**	7.0±0.0	7.0±0.0	7.0±0.0	7.0±0.0
**LTA**	420±16	1900±535	554±43	802±50
**K12-LPS**	3827±195	5483±1657	1743±361	618±43
**PG-LPS**	193±9.0	861±223	132±7.0	26±1.0

THP-1-derived CD14-high- and CD14-low-expressing (CD14^hi^ and CD14^lo^) M1 and M2 MΦs were either unstimulated (control) or stimulated with 100 ng/ml PG-LPS, 100 ng/ml *E. coli* K12 LPS (TLR4) or 10 µg/ml LTA (TLR2) for 18 hours. Endotoxin activity was investigated by TNFα secretion, presented as mean ± SD in pg/ml. Data displayed is representative of triplicate samples for n = 3 replicate experiments.

### PG-LPS and HKPG induce differential NFκB activation amplitudes in CD14^hi^/CD14^lo^ M1 and M2 MΦs

**Figure 4:**
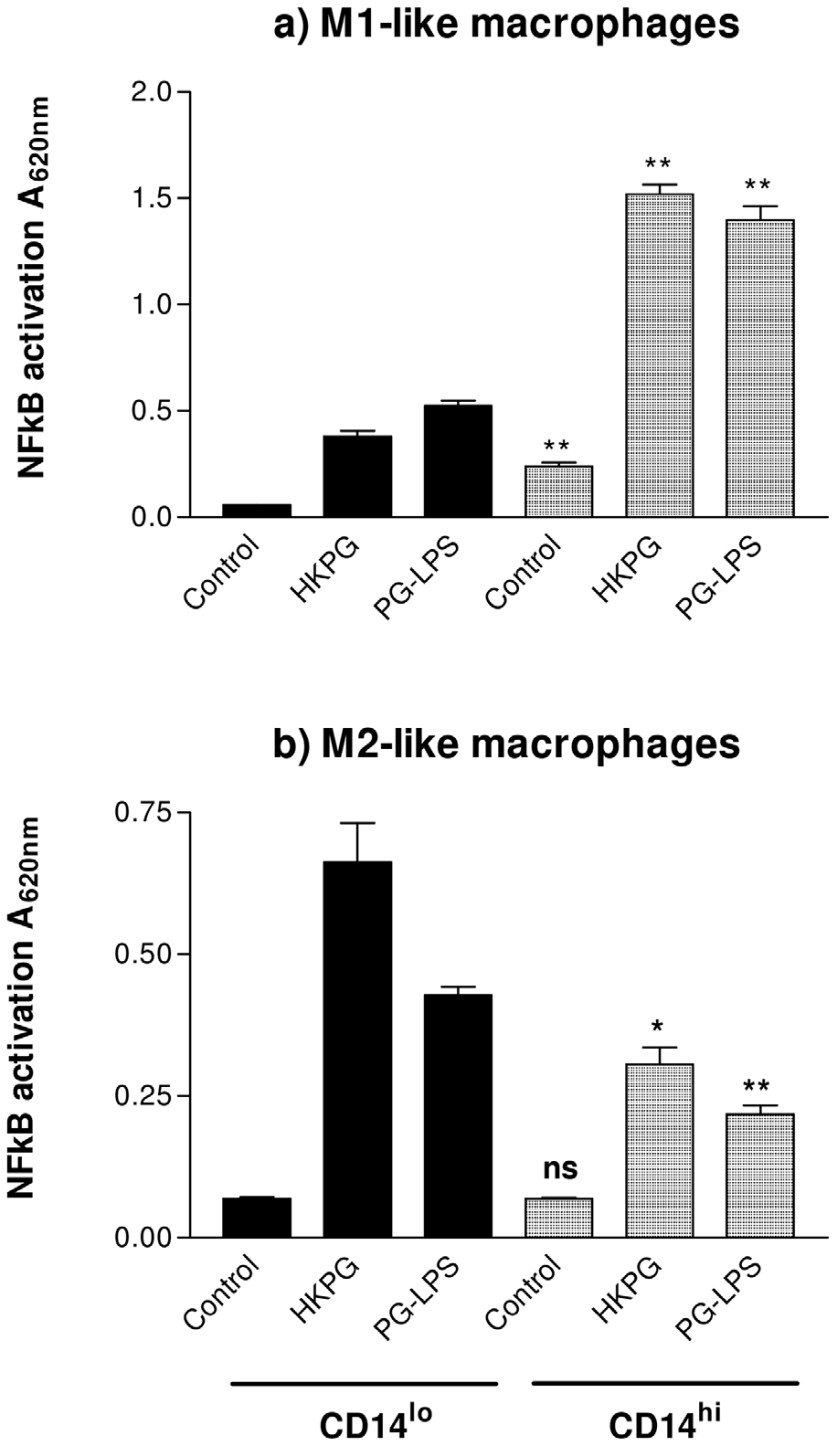
*P. gingivalis* activation of M1 & M2 MΦ induces differential CD14-dependent NFκB amplitudes. CD14^lo^ (bold) and CD14^hi^ (shaded) M1 and M2 MΦ subsets were stimulated with either 100 ng/ml PG-LPS or 1×10^7^ cells/ml HKPG. NFκB activation is expressed as the mean absorbance units A_620nm_ ± SD for M1 (a) and M2 (b) MΦ subsets. Data displayed represents triplicate samples for n = 3 replicate experiments. Significant differences in NFκB activation between CD14^hi^ and CD14^lo^ MΦs are indicated as *p<0.05, **p<0.01 and ns, not significant.

MΦ production of the pro-inflammatory cytokines TNFα, IL-1β and IL-6 has been described to be dependent on the transcription factor, NFκB. The previous section demonstrated the ability of PG-LPS and HKPG to induce these cytokines in a subset-specific manner; considering NFκB -dependence of these cytokines, it was essential to investigate whether *P. gingivalis* also induced activation of this signalling component. Indeed, M1 and M2 MΦ activation of NFκB was found to be determined by both differentiation and CD14 expression. In line with the cytokine expression data previously, CD14^lo^ and CD14^hi^ MΦs demonstrated differential NFκB activity responses when stimulated by HKPG and PG-LPS. In the case of the pro-inflammatory M1-like MΦs, M1 CD14^lo^ expressed lower NFκB activation than M1 CD14^hi^ MΦs (lower than CD14^hi^ by 75% and 62% for HKPG (p = 0.0013) and PG-LPS (p = 0.0033), respectively) (Figure 4a). The opposite trend is observed for M2-like MΦs: M2 CD14^lo^ expressed higher NFκB activation than M2 CD14^hi^ MΦs (higher than CD14^hi^ by 117% and 96% for HKPG (p = 0.014) and PG-LPS (p = 0.0053), respectively) (Figure 4b). This differential profile of NFκB activation, parallels that observed for *P. gingivalis* induction of TNFα by all of the CD14^hi/lo^ M1/M2 MΦ subsets, suggestive of a direct link between NFκB activation and MΦ production of these pro-inflammatory cytokines.

### *P. gingivalis* differentially suppresses CD14^hi/lo^ M1 and M2 MΦ NFκB activity, TNFα and the anti-inflammatory cytokine, IL-10

Previous data in this manuscript have demonstrated that PG-LPS and HKPG activation of NFκB and induction of the NFκB -dependent pro-inflammatory cytokines are differentially regulated in M1 and M2 MΦ subsets and amplitudes dependent on CD14 expression. Preliminary investigation of PG-induced tolerance/suppression demonstrated that M2 MΦs were sensitive to suppression whereas M1 MΦs were refractory. These THP-1-derived MΦ subsets are heterogenous with respect to their CD14 expression; mucosal MΦs however, demonstrate distinct CD14 profiles where tolerogenic/homeostatic mucosal MΦs are CD14^lo^ and are analogous to an M2 phenotype whereas inflammatory invasive MΦs are CD14^hi^ and resemble the pro-inflammatory M1 subset [23], [28], [29]. As a consequence of this, the ability of PG to induce tolerance/suppression in both CD14^lo^ and CD14^hi^ MΦ subsets, in the context of pro-inflammatory TNFα production and NFκB activation, was investigated.

**Figure 5:**
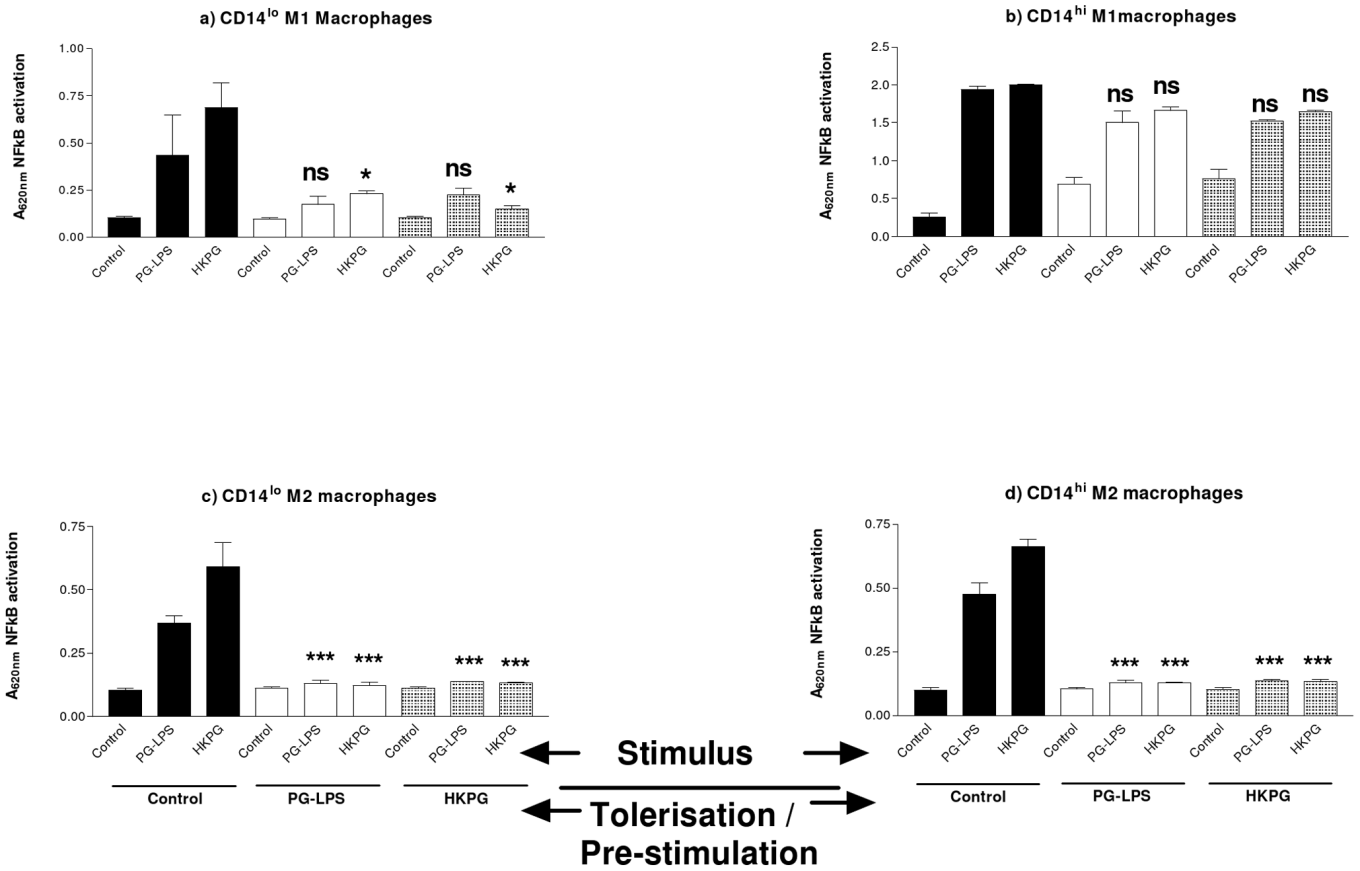
*P. gingivalis* differentially suppresses CD14^hi/lo^ M1 and M2 MΦ NFκB activity. CD14^lo^ M1 (a) CD14^hi^ M1 (b), CD14^lo^ M2 (c) and CD14^hi^ M2 (d) MΦ subsets were pre-stimulated (tolerised) with either 100 ng/ml PG-LPS (unshaded) or 1×10^7^ cells/ml HKPG (shaded) for 24 hours prior to stimulation with PG-LPS or HKPG and incubated for a further 18 hours (untolerised controls indicated in bold). NFκB activation is expressed as the mean absorbance units A_620nm_ ± SD for the CD14^hi/lo^ M1 and M2 MΦ subsets. Data displayed represents triplicate samples for n = 3 replicate experiments. Significant effects compared to the un-tolerised stimulus control (bold) for each MΦ subset are indicated as *p<0.05, ***p<0.001 and ns, not significant.

M2 MΦs were observed to be sensitive to tolerisation and cross-tolerisation by both PG-LPS and HKPG with respect to NFκB activation. CD14^lo^ and CD14^hi^ M2 MΦ NFκB activation were totally suppressed to unstimulated control levels, upon pre-treatment with these PG PAMPs (figure 5c and 5d). The pro-inflammatory MΦ subset however, was differentially sensitive to tolerance induction by PG. The CD14^hi^ M1 phenotype of MΦ, (representative of invasive, recruited pro-inflammatory MΦs) was refractory to tolerance induction by both PG-LPS and HKPG (figure 5b) whereas CD14^lo^ M1 MΦs were sensitive to pre-treatment suppression. PG-LPS stimulation control levels of NFκB activation were suppressed by 60% and 48% upon pre-treatment with PG-LPS and HKPG, respectively, whereas HKPG stimulation control was suppressed by 66% and 78%, respectively (refer to figure 5a).

**Figure 6:**
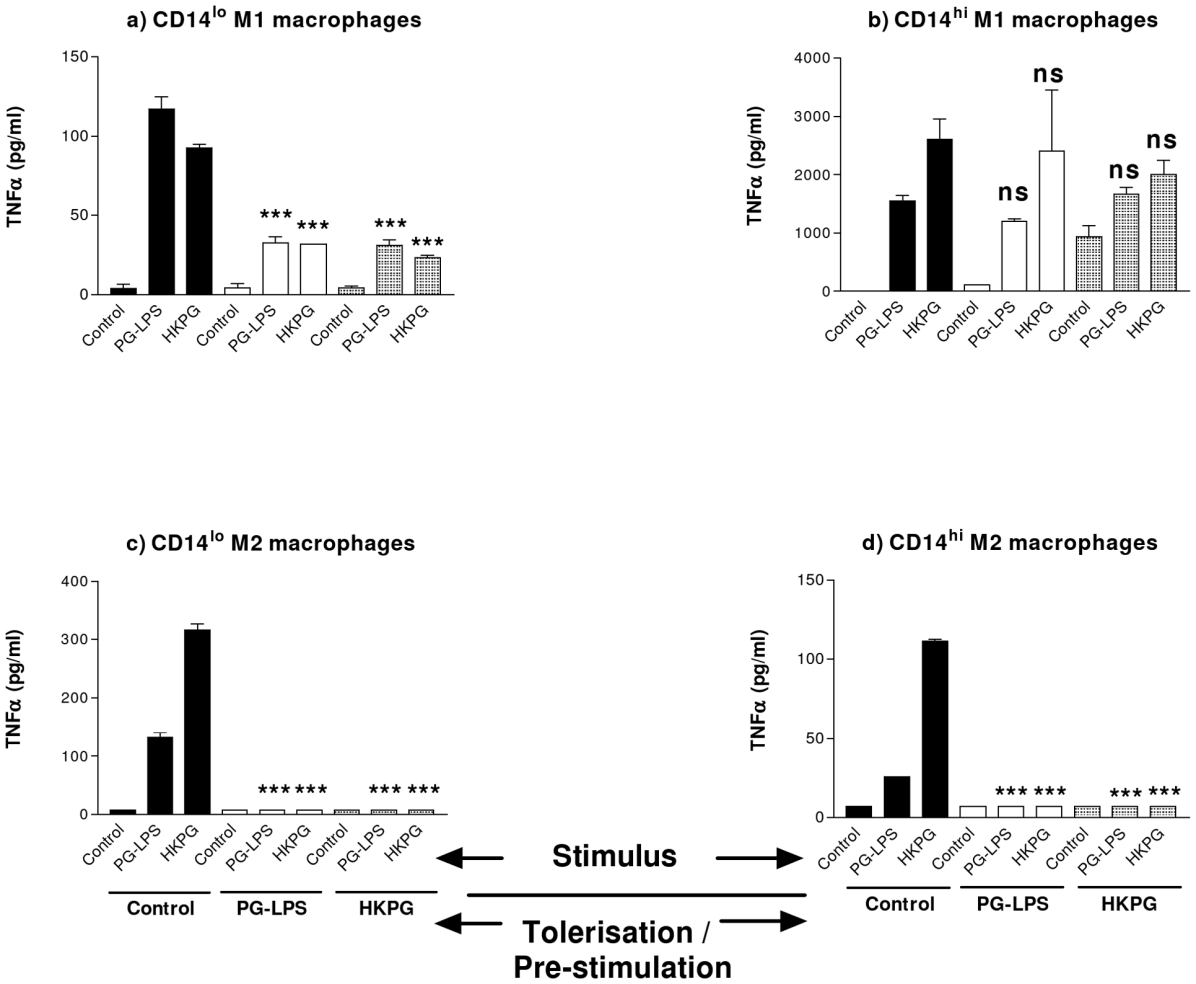
*P. gingivalis* differentially suppresses CD14^hi/lo^ M1 and M2 MΦs TNFα production. CD14^lo^ M1 (a) CD14^hi^ M1 (b), CD14^lo^ M2 (c) and CD14^hi^ M2 (d) MΦ subsets were pre-stimulated (tolerised) with either 100 ng/ml PG-LPS (unshaded) or 1×10^7^ cells/ml HKPG (shaded) for 24 hours prior to stimulation with PG-LPS or HKPG and incubated for a further 18 hours (untolerised controls indicated in bold). Pro-inflammatory TNFα cytokine production is expressed in pg/ml as the mean ± SD for the CD14^hi/lo^ M1 and M2 MΦ subsets. Data displayed represents triplicate samples for n = 3 replicate experiments. Significant effects compared to the un-tolerised stimulus control (bold) for each MΦ subset are indicated as ***p<0.001 and ns, not significant.

The induction of TNFα production by these MΦ subsets displayed the same tolerance sensitivity profile as presented with NFκB activation (refer to figure 6). CD14^lo^ and CD14^hi^ M1 & M2 MΦs exhibited different sensitivities to PG PAMP tolerisation and cross-tolerisation. In general, PG-LPS and HKPG-stimulation of TNFα production was suppressed upon pre-treatment with both the same PAMP (PG-LPS pre-treatment followed by PG-LPS stimulation and HKPG pre-treatment followed by HKPG stimulation) and the alternative PAMP (PG-LPS pre-treat, HKPG stimulus and HKPG pre-treat, PG-LPS stimulus). This suppression or tolerisation was clearly evident in both CD14^hi^ and CD14^lo^ M2 MΦs (figure 6c, 6d) and less so in the case of CD14^lo^ M1 MΦs where PG-LPS stimulation control levels were suppressed by 72% and 74% upon pre-treatment with PG-LPS and HKPG, respectively, whereas HKPG stimulation control was suppressed by 66% and 75%, respectively (figure 6a). In contrast, HKPG and PG-LPS failed to suppress HKPG and PG-LPS-stimulated TNFα production by the pro-inflammatory CD14^hi^ M1 MΦ subset (figure 6b).

**Figure 7:**
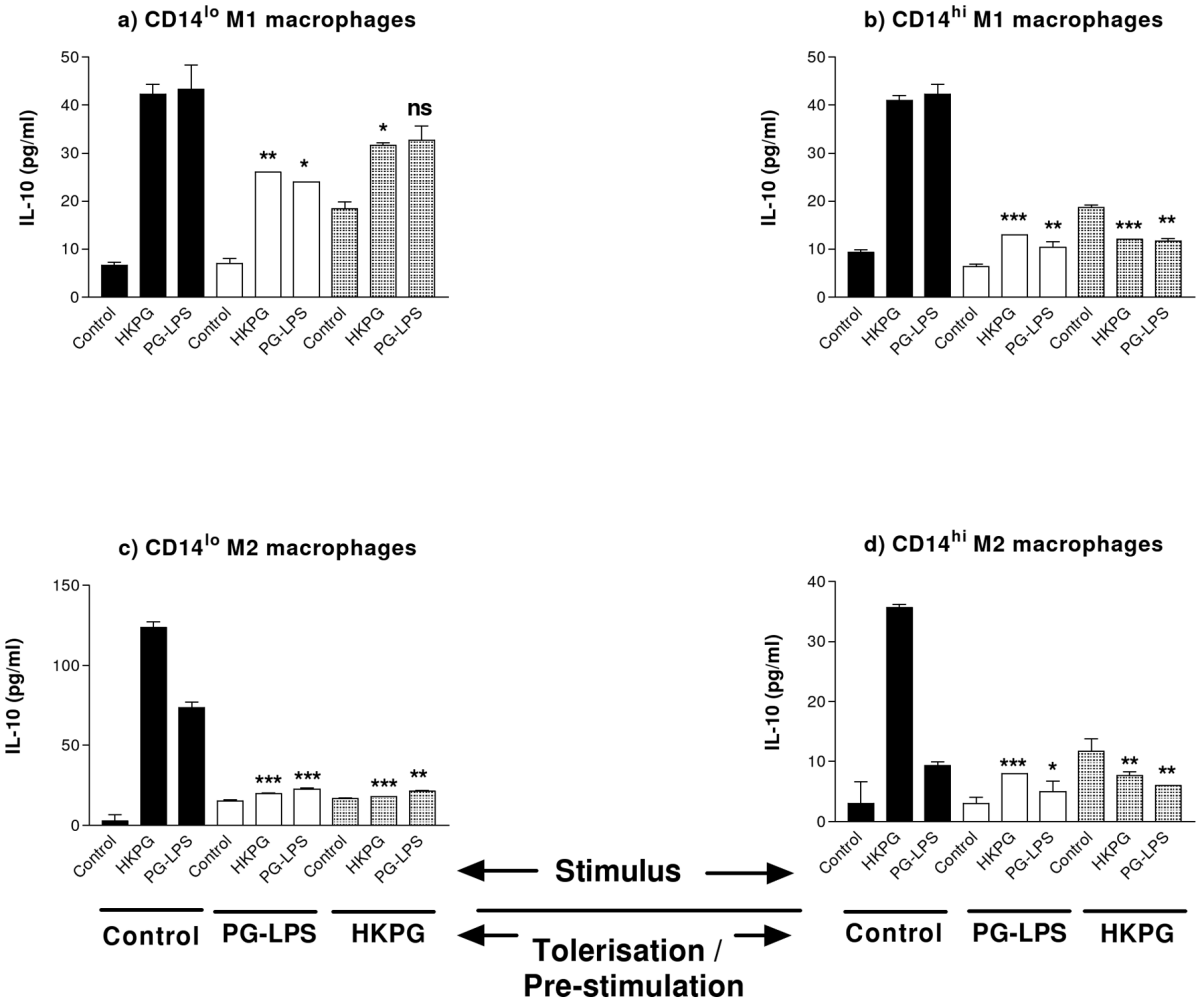
*P. gingivalis* differentially suppresses CD14^hi/lo^ M1 and M2 MΦ IL-10 production. CD14^lo^ M1 (a) CD14^hi^ M1 (b), CD14^lo^ M2 (c) and CD14^hi^ M2 (d) MΦ subsets were pre-stimulated (tolerised) with either 100 ng/ml PG-LPS (unshaded) or 1×10^7^ cells/ml HKPG (shaded) for 24 hours prior to stimulation with PG-LPS or HKPG and incubated for a further 18 hours (untolerised controls indicated in bold). Anti-inflammatory IL-10 cytokine production is expressed in pg/ml as the mean ± SD for the CD14^hi/lo^ M1 and M2 MΦ subsets. Data displayed represents triplicate samples for n = 3 replicate experiments. Significant effects compared to the un-tolerised stimulus controls (bold) for each MΦ subset are are indicated as*p<0.05, **p<0.01, ***p<0.001 and ns, not significant.

In addition, the anti-inflammatory cytokine, IL-10, also demonstrated a distinct tolerisation profile in response to HKPG and PG-LPS. Both of these *P. gingivalis* products exhibited both homo- and hetero-tolerisation of IL-10 secretion. Suppression of IL-10 was clearly demonstrated for both CD14^lo^ and CD14^hi^ M2 MΦs (figure 7c and d) where, irrespective of pre-treatment and stimulus combination, *P. gingivalis* suppressed CD14^lo^ M2 IL-10 by 70 to 85% and CD14^hi^ M2 MΦs by 34 to 78%. Interestingly, this pattern of tolerisation was extended to the pro-inflammatory CD14^hi^ M1 MΦs (figure 7b), where PG-LPS and HKPG suppressed IL-10 production between 68 to 76%, and less so the degree of suppression in the CD14^lo^ M1subset 23 to 44% suppression of *P. gingivalis* stimulus (figure 7a).

This tolerisation-sensitivity profile of these distinct MΦ subsets was reproduced when investigating other NFκB -dependent pro-inflammatory cytokines such as IL-1β and IL-6. Table 3 highlights the ability of PG-LPS and HKPG as well as the TLR2 PAMP, LTA, to tolerise and cross-tolerise these pro-inflammatory cytokines (TNFα, IL-1β and IL-6). What is evident from this table is that PG-LPS, HKPG and LTA-induced cytokines are sensitive to suppression by pre-treatment with PG-LPS, HKPG and LTA: as with figures 5 and 6, the CD14^hi^ M1 MΦ subset was found to be refractory to tolerance induction when compared to the other subsets and that there was a preferential cytokine sensitivity to suppression where, in general, TNFα was the most sensitive and IL-1β the least sensitive to suppression (refer to table 3).

**Table 3:** CD14^hi/lo^ M1 and M2 MΦ cytokines are differentially tolerised by *P.gingivalis* and LTA PAMPs.

Tolerisation signal	Stimulus	Cytokine induced	CD14^lo^ M1	CD14^hi^ M1	CD14^lo^ M2	CD14^hi^ M2
**PG-LPS**	PG-LPS HKPG LTA	TNFα	72.0±3.0 65.5±1.0 97.7±1.0	22.7±3.2 23.0±12.3 53.7±3.4	94.7±0.0 97.7±0.0 98.4±0.0	73.0±0.0 93.7±0.0 96.6±0.0
	PG-LPS HKPG LTA	IL-1β	0.0±4.5 37.0±3.3 0.0±2.0	0.0±8.1 0.0±7.5 0.0±10.7	56.4±2.1 87.8±0.3 91.2±0.2	70.6±1.2 89.3±2.1 94.7±0.3
	PG-LPS HKPG LTA	IL-6	36.6±4.4 0.0±4.3 26.4±2.1	3.0±3.5 0.0±11.1 4.1±2.9	46.2±7.1 82.2±1.0 87.6±0.2	62.4±0.5 85.9±1.0 95.2±0.1
**HKPG**	PG-LPS HKPG LTA	TNFα	73.4±2.9 74.8±1.7 91.6±1.7	0.0±7.5 23.0±9.4 0.0±2.6	94.7±0.0 97.7±0.0 98.4±0.0	73.0±0.0 93.7±0.0 96.6±0.0
	PG-LPS HKPG LTA	IL-1β	0.0±10.8 27.7±1.7 0.0±1.6	0.0±4.3 0.0±14.5 0.0±2.3	35.8±19.9 85.2±0.2 75.9±1.7	52.2±1.3 83.2±4.4 88.3±0.7
	PG-LPS HKPG LTA	IL-6	48.3±7.3 0.0±3.6 15.9±3.1	0.0±2.4 0.0±5.8 0.0±1.8	46.8±8.8 82.9±1.1 77.5±0.7	42.3±3.3 75.7±4.6 90.1±0.1
**LTA**	PG-LPS HKPG LTA	TNFα	31.5±4.9 14.0±4.1 30.2±5.0	2.9±1.6 18.3±0.8 0.8±3.9	94.7±0.0 97.7±0.0 98.5±0.0	73.1±0.0 93.7±0.0 96.6±0.0
	PG-LPS HKPG LTA	IL-1β	18.4±2.9 59.5±1.7 31.6±2.1	0.0±3.6 0.0±7.0 23.4±10.8	76.9±1.0 86.6±0.9 88.6±0.3	71.9±1.3 88.2±0.3 88.3±0.7
	PG-LPS HKPG LTA	IL-6	38.1±2.3 0.0±1.9 29.4±2.5	34.7±2.1 14.9±4.2 2.9±4.8	68.7±2.3 77.7±0.6 77.5±0.7	58.8±0.0 77.3±1.6 90.1±0.1

CD14^hi/lo^ M1 and M2 MΦ subsets exhibit a differential tolerisation and cross-tolerisation of cytokine production to the *P gingivalis* PAMPs, PG-LPS and HKPG as well as the TLR2 PAMP, lipoteichoic acid (LTA). CD14^lo^ M1, CD14^hi^ M1, CD14^lo^ M2 and CD14^hi^ M2 MΦ subsets were pre-stimulated (tolerised) with either 100 ng/ml PG-LPS, 1×10^7^ cells/ml HKPG or 10 µg/ml LTA for 24 hours prior to stimulation with PG-LPS, HKPG or LTA and incubated for a further 18 hours. Tolerisation/suppression of the pro-inflammatory cytokines, TNFα (also refer to graphs in figure 6), IL-1β and IL-6 is expressed as the mean percentage suppression ± SD of non-tolerised stimulation controls. Data displayed represents triplicate samples for n = 3 replicate experiments.

### Peptidoglycan differentially cross-tolerises *P. gingivalis*-stimulated macrophage subsets

Cross-tolerisation has been described between different microbial species, their PAMPs and the corresponding PRRs, which may have a role to play in the inflammatory process of CP, which, in addition to *P. gingivalis*, is generally driven by a collection of oral pathogens. In addition to the suggestion of cross-tolerisation exhibited between HKPG, PG-LPS and LTA and the differing suppression observed between these PAMPs in the previous table, it was desirable to investigate this process with respect to the bacterial cell wall PAMP, peptidoglycan (PGN). In contrast to PG-LPS, HKPG and LTA tolerisation, PGN exhibits a different pattern of macrophage tolerisation. PGN tolerisation, in general, resulted in a higher level of suppression of the pro-inflammatory cytokines (TNFα, IL-1β, IL-6) and the anti-inflammatory cytokine, IL-10, in all the CD14^hi/lo^ M1/M2 subsets when compared to *P.gingivalis* and LTA tolerisation. The most striking result however, was the observation that CD14^lo^ M1 MΦs were refractory (3% and 0% suppression) to tolerisation of IL-6 response, whereas CD14^hi^ M1 and M2 MΦs exhibited a high level of suppression. Finally, PGN-induced suppression of NFκB activity was weakest in both CD14^hi^ M1 and M2 macrophages (refer to table 4).

**Table 4:** Peptidoglycan differentially cross-tolerises *P. gingivalis*-stimulated CD14^hi/lo^ M1 and M2 MΦ subsets.

Tolerisationsignal	Stimulus	Mediator	CD14^lo^ M1	CD14^hi^ M1	CD14^lo^ M2	CD14^hi^ M2
**PGN**	PG-LPS HKPG	NFkB	24.8±3.8 23.4±1.5	13.3±2.1 9.0±1.7	31.8±1.8 19.6±5.7	15.5±8.2 4.4±3.4
	PG-LPS HKPG	TNFα	74.2±0.5 74.1±0.8	39.4±5.3 85.8±2.9	96.3±0.5 97.5±0.9	70.8±50.5 91.2±6.1
	PG-LPS HKPG	IL-1β	20.7±2.4 70.8±1.2	77.3±18.0 46.6±4.3	80.6±1.2 87.2±0.9	40.7±3.8 70.5±2.3
	PG-LPS HKPG	IL-6	3.3±3.1 0.0±0.0	63.8±1.3 75.2±0.4	84.9±0.5 94.7±0.3	59.7±0.8 85.6±0.9
	PG-LPS HKPG	IL-10	46.5±2.3 40.5±2.4	64.3±0.0 46.3±11.2	78.4±1.4 87.1±0.0	48.2±6.4 69.5±12.1

CD14^hi/lo^ M1 and M2 MΦ subsets exhibit a differential cross-tolerisation of cytokine production and NFκB activity to the *P gingivalis* PAMPs, PG-LPS and HKPG. CD14^lo^ M1, CD14^hi^ M1, CD14^lo^ M2 and CD14^hi^ M2 MΦ subsets were pre-stimulated (tolerised) with 10µg/ml peptidoglycan (PGN) for 24 hours prior to stimulation with PG-LPS or HKPG and incubated for a further 18 hours. Tolerisation/suppression of the pro-inflammatory cytokines (TNFα, IL-1β and IL-6), the anti-inflammatory cytokine (IL-10) and the transcription factor activity (NFκB) is expressed as the mean percentage suppression ± SD of non-tolerised stimulation controls. Data displayed represents triplicate samples for n = 3 replicate experiments.

## Discussion

This investigation has resulted in several conclusions being drawn with respect to MΦ responses to the oral pathogen, *Porphyromonas gingivalis*. Firstly, the PAMP-induced profile of pro-inflammatory cytokine production is dependent on both the route of MΦ differentiation and the level of expression of the co-receptor, CD14. In general, M1-like MΦs were characterised as TNFα^hi^, IL-1β^lo^, IL-6^lo^ whereas M2-like MΦs were TNFα^lo^, IL-1β^hi^ and IL-6^hi^. With respect to the induction of the anti-inflammatory cytokine, IL-10, CD14^hi/lo^ M1 macrophages exhibited low-level expression of IL-10 whereas higher expression was restricted to the CD14^lo^ M2 subset. Secondly, these MΦs displayed differential sensitivities to tolerance induction by both *P. gingivalis*-derived bacterial PAMPs and the TLR2 ligand, LTA ie. direct homo- and cross-/hetero-tolerance. *P. gingivalis* induced suppression of inflammatory cytokines in the CD14^lo/hi^ M2- and CD14^lo^ M1-like subsets, whereas, the pro-inflammatory CD14^hi^ M1-like subset was refractory to tolerance induction. Finally, this MΦ pro-inflammatory cytokine tolerisation profile appeared to be linked to sensitivity to suppression of the pro-inflammatory transcription factor, NFκB.

Irrespective of stimulation, the M1 and M2 subsets displayed differing cytokine effector profiles: M1 MΦs exhibited a pro-inflammatory phenotype (TNFα^hi^, IL-1β^lo^, IL-6^lo^, IL-10^lo^) whereas M2 MΦs were less inflammatory and tending to anti-inflammatory/regulatory when compared to M1s (TNFα^lo^, IL-1β^hi^, IL-6^hi^, IL-10^+^). In line with characteristic mucosal MΦ phenotypes, CD14 expression determined M1 and M2 cytokine amplitudes and NFκB activation resulting from *P. gingivalis* stimulation. CD14^hi^ M1 MΦs (representative of recruited, pro-inflammatory pathological MΦs) was described as TNFα^hi^, NFκB^ hi^ whereas the CD14^lo^ M1subset was TNFα^lo^, NFκB^ lo.^ On the other hand, CD14^lo^ M2s (representative of regulatory, anti-inflammatory mucosal MΦs) were TNFα^lo^, NFκB^ med^ and CD14^hi^ M2s were TNFα^lo^, NFκB^lo^. Contrary to our understanding of pro-inflammatory and anti-inflammatory MΦ subsets, M2-like MΦs produce higher levels of both IL-6 and IL-1β in response to PG-LPS and HKPG. These two cytokines, although thought of as pro-inflammatory, exhibit clear anti-inflammatory properties. IL-6 exerts its anti-inflammatory effects through induction of SOCS proteins and STAT-3 activation [30] and reviewed in [31]. Indeed, SOCS-3 is associated with M1 classical MΦ polarisation and is suppressive to anti-inflammatory signal and expression of IL-6 and IL-10. Conversely, SOCS-3 expression knockdown favours M2 polarisation [32]. Thus, the reciprocal relationship between SOCS-3 and STAT-3 would appear to regulate pro- or anti-inflammatory effect of IL-6 and the polarisation of MΦs between M1 and M2 effector subsets. IL-1β, on the other hand, may mediate anti-inflammatory responses via its ability to induce IL-10 expression [33]; indeed, results from this study are suggestive of a positive correlation between IL-1β and IL-10, as these cytokines are produced strongest by the CD14^lo^ anti-inflammatory/regulatory M2 macrophages. In addition, IL-1β secretion has been demonstrated to be negatively associated with the pro-inflammatory IKKβ-dependent NFκB pathway [34]; suggestive of a non-pro-inflammatory role for IL-1β and the complex wiring of the NFκB pathway in determining cell effector phenotype. Modulation of effector phenotype would thus play an important role in determining whether responses initiated in the oral mucosa are pro-inflammatory, destructive or anti-inflammatory, tolerogenic. Specific modulation of such subsets would directly affect pathogenic mechanisms associated with pathogens infecting the oral mucosa.

Mucosal MΦs are considered to exist in discrete functional subsets, governed by the environment that exists in the mucosal tissue itself. In homeostatic conditions, mucosal MΦs fail to express CD14 and express a functional phenotype resembling the regulatory, anti-inflammatory M2 subset [23], [28], [29]. Upon mucosal dysfunction, barrier breakdown and inflammatory pathological conditions, these tolerogenic MΦs change their effector phenotype to a predominantly pro-inflammatory M1-like subset. Manipulation of MΦ effector phenotype via controlling monocyte/MΦ infiltration into the mucosa, plasticity between M1 and M2 subsets, or indeed specific MΦ subset tolerance induction would be of great benefit for future therapeutic management of such inflammatory pathologies as chronic periodontitis. In the context of mucosal MΦs, whether CD14 expression is integral to tolerance induction or is just reflective of a tolerisable sensitive subset is not proven. CD14 is known to be co-expressed with both TLR2 and TLR4, both of which can be utilised by *P.gingivalis*. PG-LPS is generally recognised as transducing its signal through TLR2. Data presented in this study suggested that *P. gingivalis* and PAMPs derived from other microbes which signal through different PRRs, induce cross-tolerance, whereby peptidoglycan (which signals through NOD2) differentially tolerised both PG-LPS and HKPG (TLR2)-induced macrophage cytokines. In line with other published studies, it is probable that PAMPs such as *E. coli*-K12 LPS (gram-negative bacterial PAMP signalling through TLR4) are able to differentially suppress M1 and M2 responses to gram-positive bacteria and signals transduced through both TLR2 (homo-tolerance) and non-TLR2 (hetero- or cross-tolerance) PRRs such as TLR4 and NOD2 [35]–[37].

The fact that CD14^hi^ M1 pro-inflammatory MΦs were refractory to tolerance-induction by HKPG and PG-LPS suggested that MΦ tolerance sensitivity was, in part, dependent on CD14. This was observed for both pro-inflammatory cytokine production (TNFα, IL-1β and IL-6) and NFκB, whereas IL-10 was suppressed in these macrophages; suggesting that tolerance-induction was only partially dependent on NFκB activity and CD14 expression. Several mechanisms have been described which are involved in ET of NFκB -dependent readouts. These include the up-regulation of endogenous suppressors of NFκB activation such as SIGIRR, ST2, A20, Myd88 s and IRAK-M [9], [38]. NFκB is also important with respect to MΦ subset polarisation; IκBα over-expression resulted in M2 polarisation [19], whereas IKKβ deletion favoured M1 MΦs [20]. Thus, M1 subset polarisation is dependent on the classical p65/p50 NFκB heterodimer and M2 polarisation was found to be dependent on the alternative NFκB p50/p50 homodimer [21]. Manipulation of such classical and alternative NFκB pathways is likely to have a dramatic influence on MΦ plasticity, hence determining immune response as either pro-inflammatory/immune activatory or anti-inflammatory/tolerogenic.

Stimulation and pre-stimulation protocols investigate tolerisation by PG-LPS and HKPG, allowing the study of signals via PRRs but does not consider soluble/secreted immunomodulatory components produced by live bacteria. *P.gingivalis* secretes gingipains which are involved in endotoxin tolerance by cleavage of CD14 from the cell surface, leading to LPS hypo-responsiveness [24], either through CD14 absence from the LPS-binding receptor complex or through secreted CD14 competing for the LPS/LBP complex, hence antagonising the LPS-TLR signal, reviewed in [9]. It is probable that these gingipains may also induce ET through the shedding of PRRs such as TLR4, TLR2 and TLR5. In addition, this induction of ET may also be mediated via a protease-mediated shedding of both membrane-bound TNFα and its receptor, TNF-Rp75 [39]; hence suppression of TNFα-mediated inflammatory responses.

Chronic periodontitis is not just driven by *P. gingivalis* alone. To appreciate all the underlying pathological mechanisms, the complex interactions between the host immune factors and the microbial ecosystem of oral commensal and pathogenic bacteria requires investigation. Indeed, CP is characterised by bacterial plaque formation; it is likely these complex bacterial biofilms play a significant role in protecting pathogens from host immune responses either as a consequence of inaccessibility to damaging immune responses or through the regulation/deviation of these defences. One such intriguing pathogen response to host immunity by PG biofilms was found to be via the degredation of both pro-inflammatory (IL-1β, IL-6) and anti-inflammatory (IL-1Ra) cytokines [40]. Such an immuno-suppressive mechanism was again indicative of microbial protease activity.

The significance of these data in the context of CP is difficult to interpret. In general, any mechanism, which induces tolerance is likely to be beneficial to chronic pathologies that result from over-exuberant immune responses. These data clearly demonstrate a role for PG in tolerance induction of M2 and CD14^lo^ M1 MΦ-inflammatory mediators, whereas no suppression was observed with inflammatory CD14^hi^ M1 MΦs. This suggested some beneficial effect to the oral pathogen by failing to suppress the pro-inflammatory macrophage. Indeed, early *P. gingivalis* infection events were found to be anti-inflammatory or tolerant, enabling the pathogen to expand its numbers. This population expansion leads to an increase in inflammatory mechanisms, resulting in tissue destruction, lesions and a reduction in bacterial numbers. As a consequence of this cycling in pathogen numbers; it is likely that this relapsing/remitting chronic inflammatory disease is characterised by immunopathological mechanisms constantly switching between inflammation and tolerance/regulation. Maintenance of this chronic cycling between ET and destructive inflammation over a long period is detrimental to the host; long-term ET rendering the host more susceptible to infection (immunocompromised) and long-term inflammatory responses resulting in host tissue destruction without pathogen clearance.

In conclusion, this investigation has further characterised M1- and M2-like MΦ subsets with respect to pro-inflammatory cytokine profile upon stimulation with *P. gingivalis* PAMPs. It demonstrates a dichotomy in cytokine secretion where M1 MΦs are indeed the predominant pro-inflammatory cell. This effector response was further elucidated in the context of subsets relevant to mucosal MΦs where, in response to *P. gingivalis*, the CD14^lo^ M2 subset, representative of regulatory, anti-inflammatory cells, was indeed a low-level producer of TNFα and IL-10^+^ whereas CD14^hi^ M1 MΦs, representative of infiltrated pro-inflammatory pathological cells, were predominantly pro-inflammatory and strongly produced TNFα. This cytokine profile is likely to be as a consequence of NFκB activation, as NFκB activation profile for these MΦ subsets, closely paralleled the cytokine response. In addition, upon investigation of sensitivity of these subsets to tolerisation, it was observed that the subset least sensitive to *P. gingivalis*-induced suppression of pro-inflammatory cytokines and NFκB activation was the inflammatory pathology-related subset, CD14^hi^ M1. This would suggest that such mechanisms of ET may be beneficial for survival and immunopathological mechanisms driven by the pathogen. To conclude, any future manipulation of MΦ subset suppression can only realistically be employed upon a full understanding of the immunopathological mechanisms behind such relapsing/remitting diseases as CP and by considering; who is tolerance induction of benefit to….host or pathogen?
